# Performance of a wearable acoustic system for fetal movement discrimination

**DOI:** 10.1371/journal.pone.0195728

**Published:** 2018-05-07

**Authors:** Jonathan Lai, Richard Woodward, Yuriy Alexandrov, Qurratul ain Munnee, Christoph C. Lees, Ravi Vaidyanathan, Niamh C. Nowlan

**Affiliations:** 1 Imperial College London, Centre for Fetal Care, Queen Charlotte’s and Chelsea Hospital, Imperial College Healthcare National Health Service Trust, London, United Kingdom; 2 Department of Mechanical Engineering, Imperial College London, London, United Kingdom; 3 Department of Bioengineering, Imperial College London, London, United Kingdom; 4 Department of Development and Regeneration, KU Leuven, Leuven, Belgium; Bloomlife, NETHERLANDS

## Abstract

Fetal movements (FM) are a key factor in clinical management of high-risk pregnancies such as fetal growth restriction. While maternal perception of reduced FM can trigger self-referral to obstetric services, maternal sensation is highly subjective. Objective, reliable monitoring of fetal movement patterns outside clinical environs is not currently possible. A wearable and non-transmitting system capable of sensing fetal movements over extended periods of time would be extremely valuable, not only for monitoring individual fetal health, but also for establishing normal levels of movement in the population at large. Wearable monitors based on accelerometers have previously been proposed as a means of tracking FM, but such systems have difficulty separating maternal and fetal activity and have not matured to the level of clinical use. We introduce a new wearable system based on a novel combination of accelerometers and bespoke acoustic sensors as well as an advanced signal processing architecture to identify and discriminate between types of fetal movements. We validate the system with concurrent ultrasound tests on a cohort of 44 pregnant women and demonstrate that the garment is capable of both detecting and discriminating the vigorous, whole-body ‘startle’ movements of a fetus. These results demonstrate the promise of multimodal sensing for the development of a low-cost, non-transmitting wearable monitor for fetal movements.

## Introduction

Fetal movement (FM) has long been of interest to the medical and scientific communities as a measure of fetal health and of neurobehavioral development. Fetal movement can currently only be quantified using ultrasound [1, 2] or MRI scanning [3], both of which are expensive, can only be performed for short windows of time, and must be undertaken in a clinical setting. Maternally sensed decreased FM are a common reason for consultation with obstetric services [4], but maternal perception of movement is highly subjective and patient-dependent [5]. It has been reported that 22–25% of women perceiving decreased fetal movements have poor outcomes at birth, such as preterm or small for gestational age births [4, 6]. Decreased fetal movements may be “a warning sign of impending fetal death” [7], with up to 57% of stillbirths in one particular study [8] being preceded by decreased fetal movements. Meanwhile, incorrectly perceived decreased FM can lead to high anxiety in an already anxious patient group. “Kick-counting”, where mothers record fetal movements every day [9] used to be the accepted norm in some countries, but the process was largely discontinued, due to the results of a major study published in the Lancet which showed that by the time the mother perceived a consistent decrease in movements, it was usually too late to save the baby [10]. However, the conclusions of the Lancet study have been disputed in more recent reviews [11, 12]. A recent guideline from the Royal College of Obstetricians and Gynaecologists [7] highlighted the lack of robust “studies on fetal activity patterns” and the lack of a universally accepted definition of reduced fetal movements. A wearable, low-cost and non-transmitting modality capable of sensing fetal movements over extended periods of time would be extremely valuable, not only for monitoring individual fetuses, but also for establishing normal levels of movement in a large population.

A small body of literature has explored the development of wearable sensors for fetal movements, and their validation against concurrent ultrasound, the gold-standard for quantifying fetal movements. Girier et al [13] used a single accelerometer placed on the abdomen and a threshold signal processing method to detect fetal movements. The system was validated during concurrent ultrasound scanning of 27 women with a mean gestation of 35 weeks. Average true and false detection rates were 48% and 40% respectively, and output signals were found to be corrupted by maternal activity, such as breathing and coughing. Ryo et al. [14] used capacitive accelerometers to monitor fetal movements (based on technology originally described in [15]), and compared accelerometer signals with concurrent ultrasonography in 45 exams performed on a cohort of 14 pregnant subjects. Fetal movements observed in the ultrasound scan were classified as gross trunk movement, isolated limb movement or breathing movement. Ten-second-long epochs of time were used as the unit of comparison, where output signals were manually compared with observed movements, For gross movements, there was 38.5% positive agreement from 20–29 gestational weeks and 23.5% positive agreement from 30–39 gestational weeks, and very low positive agreement for limb (5.0–13.3%) and breathing (4.3–22.8%) movements. In a small study of three women, Mesbah et al. [16] compared the performance of tri-axial accelerometers combined with a root-mean-square detection method, and found agreement rates with ultrasound varying from 50 to 76% between subjects. While the accelerometer-based systems described in these studies have had varying success in monitoring FM, they cannot distinguish maternal movement from fetal activity, and these technologies are not in widespread use in clinics or at home. Additional sensor modalities and reference data would be of significant benefit to the field [17]. A need therefore remains for a reliable and accurate wearable sensor systems and appropriate signal processing algorithms for monitoring FM.

In this study, we investigate if acoustic sensing can be used to identify, and discriminate between, different types of fetal movements. Acoustic signals have been reported as being “valuable for fetal surveillance … but hidden by maternal and environmental noises” [18]. We describe the design of a sensor system incorporating multiple acoustic sensors [19], combined with an inertial measurement unit which facilitates removal of artefacts due to maternal movements, and propose novel signal processing algorithms. Finally, we validate the hardware and software by comparing the movements detected by the sensor system and signal processing methods with the number and types of movements observed during concurrent ultrasound scanning in 44 pregnant subjects.

## Materials and methods

### Hardware

Our research team has designed and, through iterative prototypes, optimised a new sensor system embedded in a maternal support band. The package consists of a custom-made inertial measurement unit (IMU) that simultaneously fuses input from 8 acoustic sensors (which detect vibrations resulting from fetal movement) and a tri-axial accelerometer (which detects maternal motions). A summary of the system follows.

#### Acoustic sensors

**Fig 1:**
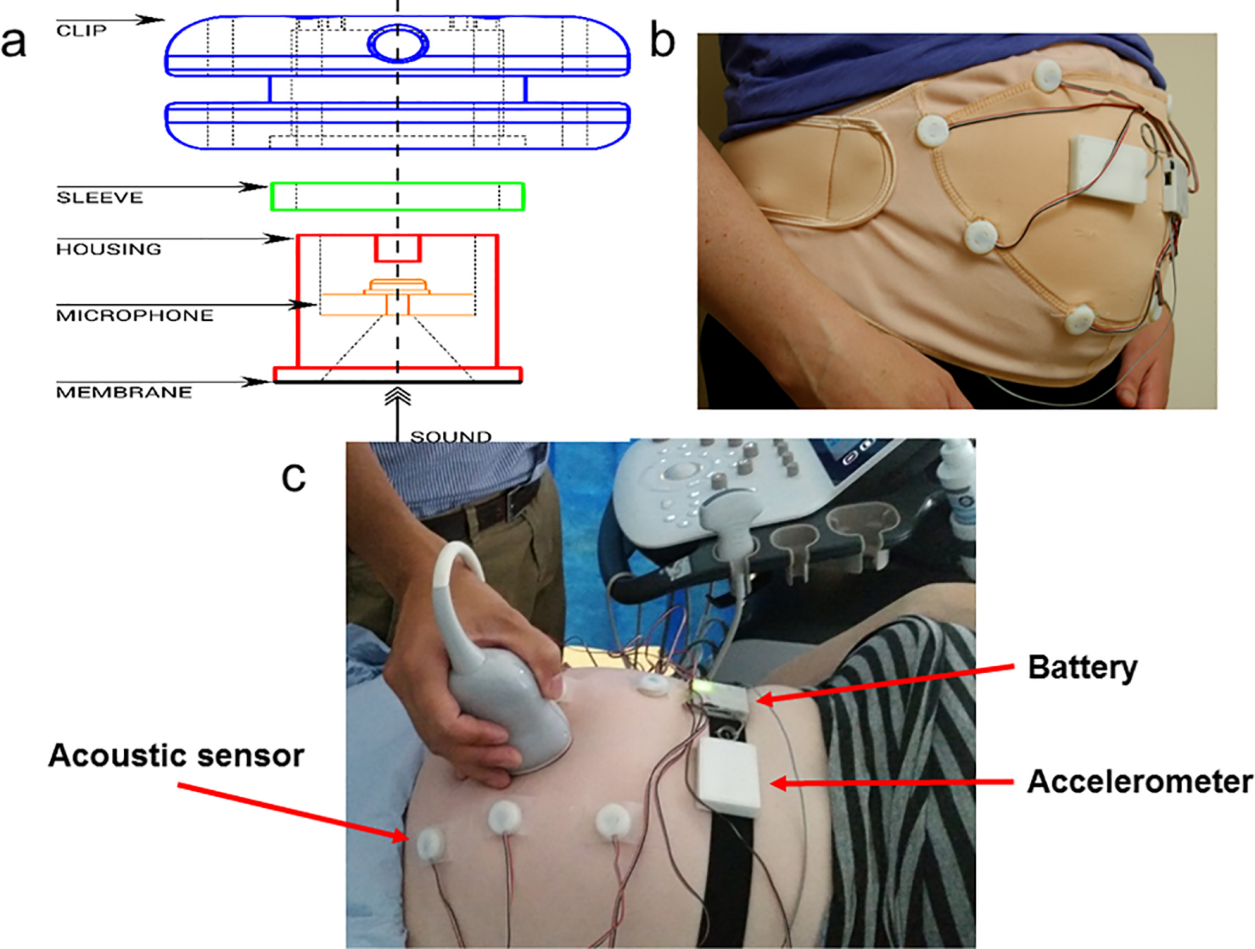
Proposed fetal movement monitor and experimental set-up. (a) acoustic sensor design; (b) wearable version of sensor system; (c) experimental set-up with ultrasound-compatible version of sensor system, enabling field of view of ultrasound probe.

The acoustic sensor consists of a diaphragm covering a sealed chamber dimensioned to capture low frequency vibrations. While accelerometers have been used for fetal monitoring and other physiological acoustic sensing [20], they potentially suffer from signal occlusion from maternal motion picked up by the motion sensor. The sensor suite introduced in this work is a modified version of a system introduced to capture vibrations associated with muscle contraction [21]. A sensor detects pressure change from the base of a sealed chamber. The chamber is covered using a piece of Mylar, and a microphone (Knowles SPU1410) positioned at the opposite end of the chamber records the pressure change when the membrane is disturbed. When the device is placed on the abdomen, fetal movements produce a low frequency vibration that propagates through the membrane and creates a pressure difference within the chamber. Fig 1A shows a schematic of the sensor that measures 21x9 mm (ØxH). The Mylar membrane is wrapped around the base of the device and is held in place using a sleeve and friction compression of the fitted parts.

#### Inertial measurement unit

Our custom made IMU (45x30x9 mm (LxWxH)), can be seen in Fig 1B & 1C in the centre of the worn device with an array of acoustic sensors. It consists of a ±16 g tri-axis accelerometer (Analog Devices ADXL345). The microcontroller’s analogue to digital converter allows sampling from up to 8 acoustic sensors in this application. Both inertial and analogue to digital converter data are stored on a removable micro SD card for offline data processing, and thus no wireless transmission is necessary. Real-time date and time recording is possible due to a 32.768 kHz crystal oscillator (ECS Inc. ECX-31B) which can be time-stamped to the data before processing.

#### Wearable and ultrasound versions

Fig 1 shows the wearable monitor. Fig 1A shows the acoustic sensor, while the physical layout of the wearable version of the system is shown in Fig 1B. The system developed for the ultrasound tests (in which the maternal support band is removed, and acoustic sensor arrangement is adjusted to maximise the available field of view of the ultrasound probe) is shown in Fig 1C. Due to the necessity of leaving sufficient field of view for the ultrasound probe, only six acoustic sensors were used in the ultrasound version of the system, instead of the maximum possible eight.

### Validation tests

44 women attending for maternal outpatient hospital visits or who were inpatients at Queen Charlottes and Chelsea Hospital (London, UK) were recruited. Women with a live singleton pregnancy between the gestations of 24+3 to 34+6 weeks were invited to participate and gave written consent. The study was approved by the Research Ethics Committee (Ethics reference 15/LO/0030), and the Joint Research Compliance Office, Imperial College (Reference 14lC234). 10 women were recruited for the pilot phase of the project to examine feasibility. These data are not reported. 52 women were recruited for this project, however 8 were not analysed due to missing data leaving 44 for analysis. Recruitment occurred from June to October 2015. No subjects voluntarily dropped out of the study.

Each mother underwent a trans-abdominal ultrasound. Following standard fetal health assessments (all of which were normal), the ultrasound version of the sensor system was put in place. The belt containing the inertial measurement unit and battery was placed around the mother’s waist, above the bump, and the six acoustic sensors were affixed using surgical micropore tape in a grid arrangement, standardized using a paper template. The ultrasound probe (GE Voluson S8) was positioned to provide the best view of the fetus, and movements were monitored for roughly twenty minutes per subject. During this time, fetal movements were quantified under three categories; general (whole body movements), startles (quick generalised movement, lasting about a second) and breathing. If the fetus changed position, leading to a sub-optimal view for the ultrasound probe, the probe was moved and the timing of which was noted in the records. The occurrence of a fetal movement and its category, or any movements of the probe, were recorded using a time-synchronized software by a second researcher. All ultrasound assessments were performed by the same experienced physician (JL). Spearmann correlation tests were performed on the relationship between the number of different types of fetal movements and maternal and fetal factors (including: gestational age, body mass index, deepest vertical pool of amniotic fluid, estimated fetal weight (Hadlock algorithm), middle cerebral artery pulsatility index, umbilical artery pulsatility index).

### Software: Signal analysis

A complete mathematical signal processing architecture and software system to isolate fetal movement and discriminate signals indicative of different movement types was developed. The algorithms were implemented in MATLAB (MathWorks, USA) with a full graphic user interface (GUI) allowing the data to be loaded, assessed and analysed, and the results to be visualized. Algorithms and software were developed and implemented in ‘sensor neutral’ manner, such that other sensing modalities may be introduced into the analysis with minimal effort. This software, with the full dataset, is freely available as open source software. S1 File outlines the mathematical architecture and software implementation for the analysis and provides links for downloading and using the package.

#### Segmentation

Signals gathered from the sensor system were first pre-processed using comb-notch filtering to eliminate minor interference from acquisition electronics. The segmentation of temporal intervals of activity, or “regions of interest” (ROIs), from acoustic and IMU channels was evaluated by signal-to-noise analysis. A six-step segmentation process was developed consisting of signal averaging, de-noising, artefact removal, removal of interference from maternal movement, and fusion of all acoustic channels into a *Region of Interest* (ROI) vector consisting of ‘candidate movements’. The segmentation procedure is described in detail in the Supporting Information (S1 File).

#### Detected signal matching

Following segmentation, it was necessary to correlate each candidate movement with the physician’s annotations to gauge the accuracy of the sensor system for the three different movement types (“breathe”, “general”, and “startle”). The detection rate of the sensor system was calculated for each subject by comparing a timeline of the doctor’s annotated (observed) movements and the timeline of candidate movements (ROIs segmented from the acoustic sensor channels as described in ‘Segmentation’ above). To estimate the number of “detected ROIs” (those movements both observed by the doctor and detected by the sensor system), every doctor’s annotation was projected as a time window on the segmentation (detection) map. This time window was created by extending the annotation by 3.5s into the past and 1.5s into the future to account for reaction time, and the duration of a movement. Existing literature has classified movements with windows as large as 10 seconds [14], which was judged to be too long and potentially overly favourable to the sensor. Five seconds was chosen as an interval based on review of these practices and in consultation with examining physicians as to their own identification. Several movements occurring within a 5 second window were classified as a single movement, as long as the movement type observed remained the same. Windows of 3.5 seconds preceding and 1.5 seconds following callout were chosen due to the lag in the observation, notification and recording of the movement. Since the movement itself, of course, precedes the call-out, the time window was shifted. For each annotation (observed movement), if this window intersected with the timeline of candidate movements, the corresponding annotated ROI was considered “detected”, and was associated with the type of the annotated movement for discrimination purposes.

#### Discrimination

Our final goal was to determine if the signal properties of movement types differ enough to be able to discriminate between different movement types. The signals were transformed in a way similar to that of segmentation described earlier, except that for this part of the analysis, the absolute value of the data was not taken, to avoid loss of signal information. For each candidate movement which overlapped with a doctor’s annotation (identified as described above), the ROI from the acoustic sensor channel with the highest signal to noise ratio was selected for further analysis. The following feature vector components were calculated for each of the selected ROIs: 1) Duration, 2) Energy, 3) Entropy, 4) Six selected wavelet energy coefficients, and 5) Three frequency window energy ratio quantifiers (1.1–1.5Hz, 2.2–3Hz, 6–9Hz).

In order to assess the ability of the derived feature vector components to discriminate between different types of fetal movements annotated by the physician (e.g., to discriminate between startle and general movements), standard confusion matrices were calculated [22] using several supervised classification methods. Dimensionality reduction was first performed using a principal component analysis (PCA) coordinate transform [23]. Further classification methods used only the first three PCA components of this transformed data, to ensure numerical stability. The confusion matrix calculations were implemented for supervised classification by applying the “take one out” (“jackknife”) method. In this method, the feature vector of the ROI was taken out of the whole feature extraction data and considered as unclassified data, while the rest was considered as training data. Then, the result of the unclassified data for recognition was used to update the confusion matrices, and the whole procedure was repeated for all feature vectors. To create corresponding trained classifiers, a k-nearest neighbour (kNN) supervised classification method was used [22]. This procedure was repeated three times, to assess discriminatory capacity between startle and general, startle and breathe, and breathe and general. Quadratic discriminant analysis (QDA) and linear discriminant analysis (LDA) were also applied, for comparison with kNN.

## Results

**Fig 2:**
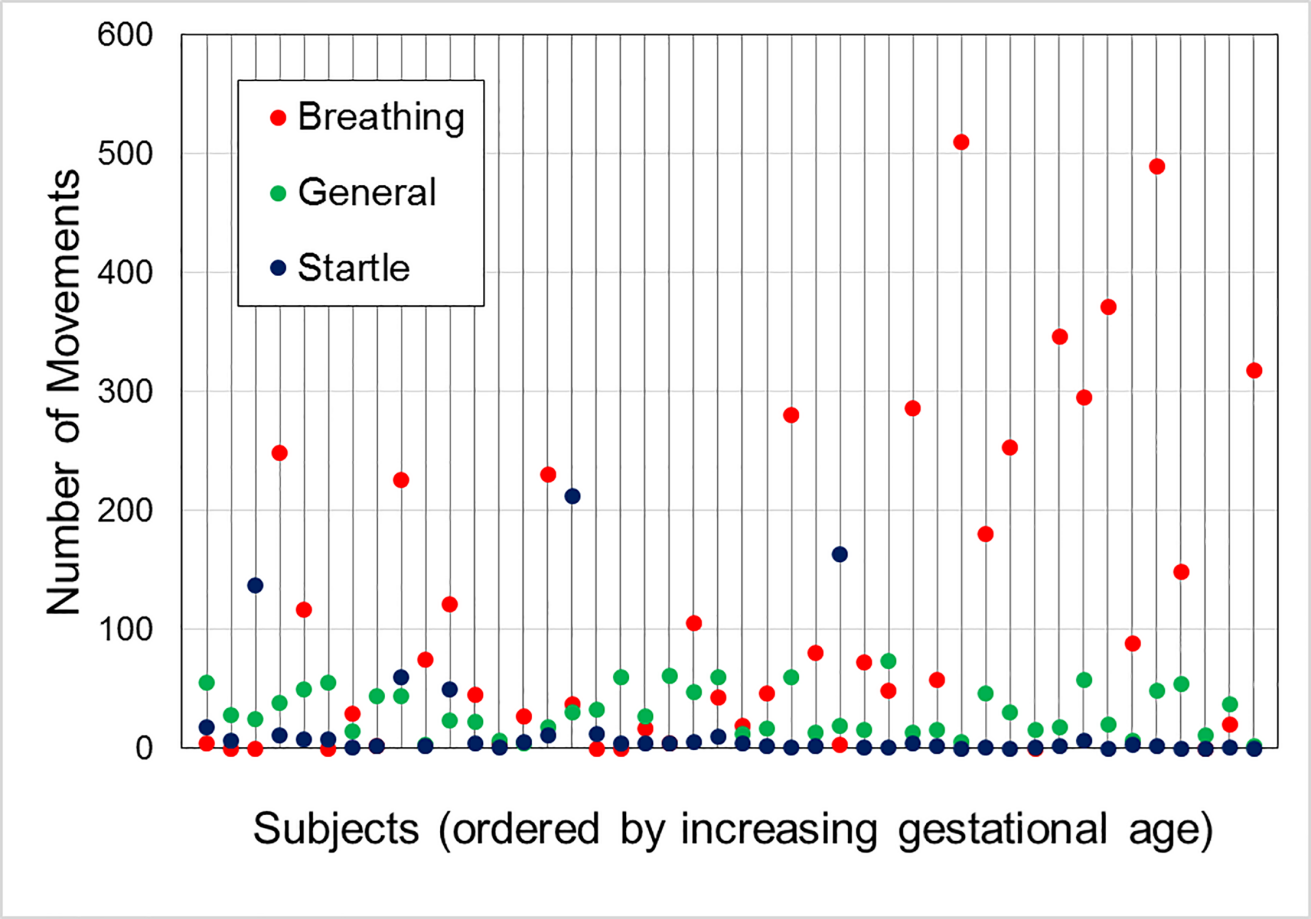
Number of breathing, general and startle movements observed during each ultrasound scan, with each vertical line representing one test subject. Subjects are ordered by gestational age at time of scan, with the youngest (25+3 GW) being at the left and the oldest (34+6 GW) at the right. Breathing movements were the most commonly observed overall, and startle movements the least common. There was no evidence of a change in the frequency of general or startle movements with gestational age, while breathing movements were correlated with increasing gestational age.

A total of 900 minutes (15 hours) of data for concurrent ultrasound scanning and sensor testing was collected for the 44 pregnant women, with each woman undergoing only one test. The average gestational age was 31 gestational weeks (range: 25+3–34+6). During this time, a total of 780 startle movements, 1354 general movements and 5251 breathing movements were observed. There was a large variation in the occurrence of different movement types between subjects, as shown in Fig 2. Only a small number of subjects had large numbers of startles (Fig 2), with only five subjects exhibiting more than 20 startle movements over the course of the scan. Three of these five exhibited over 100 startles, which (as noted by the sonographer) were due to fetal hiccups. There were no apparent effects of gestational age on the incidence of startle or general movements, but there were increasing breathing movements with gestational age (Spearman’s Correlation coefficient R: 0.395, p<0.01). A spreadsheet of data supporting this article has been uploaded as part of the Supporting information (S1 Table).

### Detection

**Fig 3:**
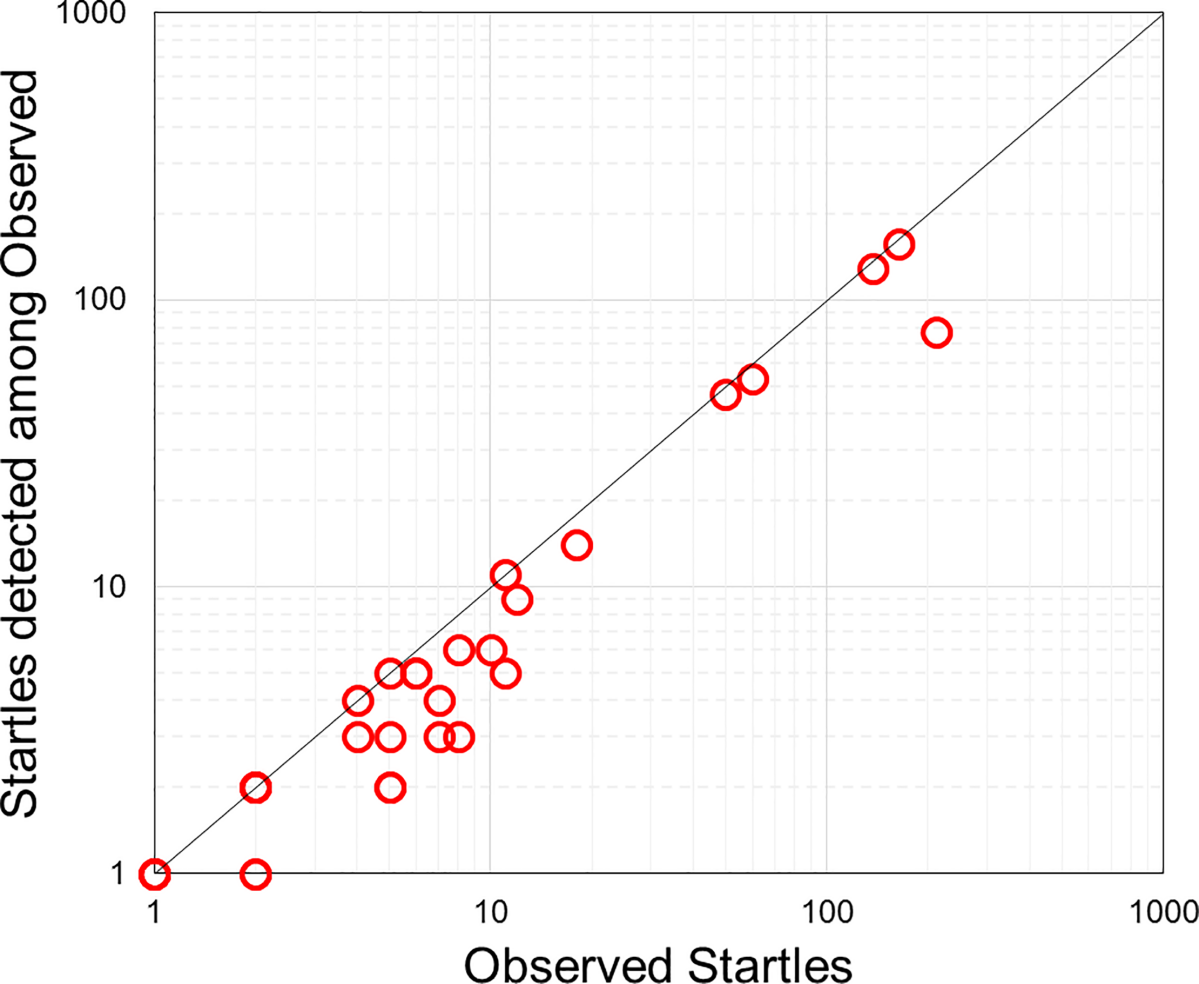
Startle movements were the most reliably detected by the sensor system, with 78% of total observed startle movements across all patients being detected. Each point represents one patient in which there was at least one startle movement observed and detected (n = 23). Log scale.

**Fig 4:**
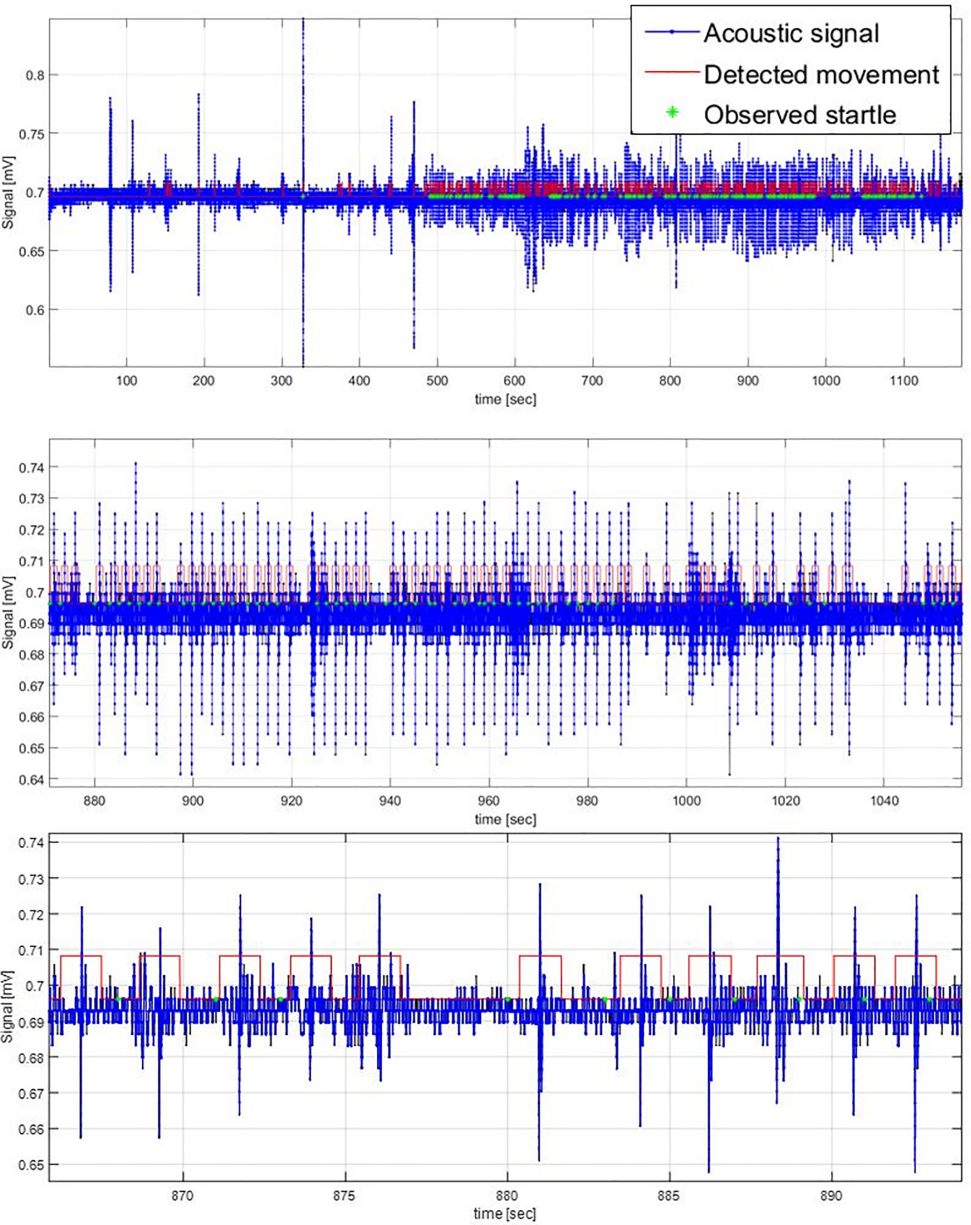
Individual sensors show promising evidence of detection of startle movements in cases of high fetal activity. Sample outputs from individual sensors from three different subjects shown: blue is unprocessed acoustic signal, red indicates movements detected by sensor system, green indicates observed startle movements.

**Fig 5:**
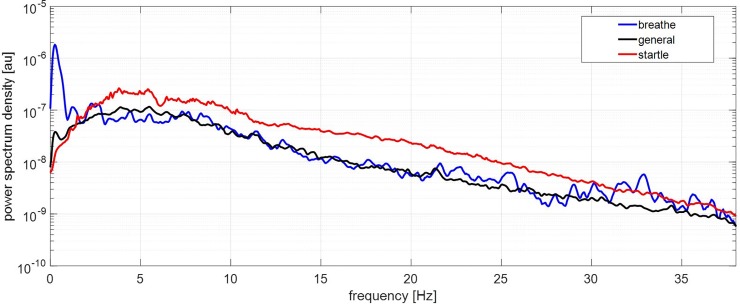
Average frequency spectra of breathe, general, and startle fetal movements as recorded by the FMM. Spectral density is concentrated under 10 Hz for all 3 movements with later peak and lower attenuation for startle movements.

Using the signal processing steps described, the sensor system detected a high proportion (78%) of all startle movements, as illustrated for individual subjects in Fig 3. Successful detection of startle movements is emphasised when looking at outputs from individual sensors in subjects in which a high number of startles occurred, as shown in Fig 4. The three examples shown exhibit a clear correlation between signal activity (in blue), observed startle movements (in green) and detected movements (in red) (Fig 4). We found that detection of fetal movements appears to be highly dependent on the location of the kick and its proximity to the nearest sensor, as most of the other acoustic sensors for the scans shown in Fig 4 did not show any obvious overlap between observed startle movements and the processed signals. Fig 5 shows the average frequency spectra of breathe (in blue), general (in black), and startle (in red) movements as recorded by the FMM. Spectral density is concentrated under 10 Hz for all 3 movements. Startle movements exhibit higher power ranges than general and breathing movements (Fig 5).

Detection rates for general and breathing movements were lower than for startle (53% and 41% respectively), and when the data for each subject was scrambled (by randomising the timeline of candidate movements), and the same analysis repeated, the percentage detection rates for general and breathing movements were similar to the original data (39% and 44% respectively). In contrast, the startle detection rate was substantially reduced (to 46%) for the scrambled data. Some types of general movements may have been detected, but the variance in this movement category made classification challenging. The results indicate that the sensor system is successfully able to detect startle movements, but is not currently sensitive to breathing or general movements.

The ratio of candidate movements identified by the sensor system to the number of movements seen was, on average across all scans, 2.4. However, the variance between scanning sessions was large, with the ratio of predicted movements to movements seen ranging from of 0.07 to 15.1. This means that the current version of the sensor has a false positive rate that varies substantially between tests. The scans with a very low ratio of predicted to seen movements were accompanied by a very high number of breathing movements, emphasising our finding that the sensor system is not sensitive to breathing movements. When a startle movement not seen by the doctor is detected, it is impossible to determine if this is due to the sensor system picking up some breathing and general movements (for example, the more vigorous general movements may be detected), recording interfering signals from the mother (e.g. digestive activities or slight movements not recorded from the IMU), interference from the surrounding environment, the examiner brushing against or tapping the sensors, or some combination of these stimuli.

### Discrimination

In order to assess the capacity of the system to distinguish between physician identified breathe, startle, and general movements, each segmented candidate movement was contrasted to the timeline of observed movements. There were 649 total candidate startle movements matching physician-identified startle movements, 1100 matching breathe movements and 1010 matching general movements. Candidate movements that matched an observed movement were then partitioned into training and test data sets, and a k nearest neighbour classifier was applied to correlate each candidate movement to the movement identified by the examiner. When distinguishing between breathing and startle, or between general and startle, the discrimination criteria were capable of correctly predicting the majority of the categorised movements. Tables 1, 2 and 3 present confusion matrices describing the success of discrimination criteria in predicting types of fetal movement. In each table, the row indicates the movement type observed by the physician, and the percentage in each cell indicates how successful the classifiers were in correctly discriminating between types of fetal movements. In Table 2, for example, of the 1010 total general ROIs, the FMM correctly identified 72.1% as a general movement and incorrectly identified 27.9% as a startle movement. The diagonal of each table, thus gives the correct rate of identification for each comparison. As shown in Table 3, it was not possible to effectively discriminate between general and breathing movements. In summary, startle movements can be distinguished from breathing or general movements based on properties of their signal detected by the sensor system, while discrimination between general and breathe movements cannot be reliably performed.

**Table 1:** Breathe vs Startle Movement. Confusion matrix outlining results for Breathe vs Startle movement discrimination.

	Breathe	Startle	Total
Breathe	66.60%	33.40%	1100
Startle	31.90%	68.10%	649

**Table 2:** General vs Startle Movement. Confusion matrix outlining results for General vs Startle movement discrimination.

	General	Startle	Total
General	72.10%	27.90%	1010
Startle	34.80%	65.20%	649

**Table 3:** Breath vs General Movement. Confusion matrix outlining results for Breathe vs General movement discrimination.

	Breathe	General	Total
Breathe	54.10%	45.90%	1100
General	45.60%	54.40%	1010

We also note that similar accuracy also achieved with quadratic discriminant analysis (QDA) and linear discriminant analysis (LDA) (data not shown).

## Discussion

We have demonstrated that an acoustic sensor system combined with accelerometers is sensitive to fetal startle movements, has the ability to discriminate between startle movements and other forms of activity, and can effectively eliminate artefacts due to maternal movement. The system is non-transmitting and would therefore be safe for use over extended periods of time.

There are some limitations to the system in its current form. The acoustic sensors have been designed for very high sensitivity, which is inevitably accompanied by susceptibility to noise. In its current form, we are not yet able to predict the level of fetal activity based on the outputs of the system alone, due primarily to a false positive rate that varies substantially between scans, which is likely a consequence of the sensitivity of the acoustic measurement. Detection is made more challenging by the fact that only one or two of six sensors are likely to register a short, vigorous movement. Over detection of movements could potentially lead to a misdiagnosing positive fetal health.

In order to increase the specificity of the system, we plan to combine acoustic sensors with accelerometer and/or pressure-based sensors in order to obtain a fetal movement monitor which is both sensitive and specific. Ideally, a combination of sensors could isolate the occurrence of a movement, which could then be isolated or discriminated in a manner similar to the results in this investigation. Future work will assess the optimal number, placement and arrangement of acoustic sensors to optimise detection, discrimination and accuracy. In common with the results of Ryo et al. [14], our study indicates that fetal breathing movements, and movements which do not involve the whole body, are not easy to detect. While our system was capable of discriminating potential breathing or general movements from startle movements, consistent detection was not possible, even with the improved sensitivity of acoustics as compared with accelerometers. Based on these results, we believe it is highly unlikely that any non-transmitting wearable sensor will be able to detect fetal breathing movements.

## Conclusions

There are several key advances made in this work in comparison with previous studies on comparing the performance of wearable fetal movement sensing technologies against concurrent ultrasound. Firstly, in demonstrating that acoustic signals can be used to detect vigorous fetal movements, we have introduced an entirely new sensing modality to measure fetal movement. Secondly, we have combined acoustic and accelerometer sensing to successfully discriminate fetal and maternal movements, enabling detection of fetal movements when the mother is active. Furthermore, we have introduced a clinical testing protocol that will serve as a basis for future studies of fetal movement monitors or sensors, which has been executed on the largest patient cohort to date. Finally, we have developed a cohesive signal analysis architecture to fuse information from multiple sensors and isolate fetal movement. In its current state, our signal analysis methodology has demonstrated the capacity to detect fetal movements, remove potential for artefacts due to maternal movement, and identify particular movement patterns. The signal processing techniques developed have also made it possible to discriminate between startle movements and other types of fetal movements. The entire architecture has been implemented in an open source software package with a full graphic user interface that can be freely used and adapted by others developing similar sensors systems (see S1 File).

The significance of this study is that it demonstrates, for the first time, that an acoustic sensing modality, in combination with advanced signal processing techniques, is capable of detecting startle movements, and is also capable of discriminating startle movements from signals associated with other signals picked up from the abdomen, which could be due to other fetal movements or due to (for example) maternal digestion. It is not surprising that startle movements were found to be the most reliably detected, as these movements are the most vigorous and the most likely to impact forcefully on the uterine wall. These are likely to be the movements that the mother is most likely to feel herself, and in future studies, we will compare movements detected by the sensor system against maternal sensation. There is substantial value in being able to objectively quantify the number of vigorous fetal movements, as when a woman is busy, she may not notice or remember how many times she felt movements.

In summary, we demonstrate that a novel acoustic sensor is capable of detecting, and discriminating, fetal startle movements, in a multi-sensor system in which maternal movements do not interfere with detection. We have also designed and implemented a signal analysis architecture in software for processing of any sensor system for fetal movement monitoring, and made it available to the general public. Therefore, this study represents a significant development towards a low-cost, non-transmitting, and wearable technology to monitor fetal movements. Commercial applications being pursued include clinical and consumer devices [19].

## Supporting information

S1 File Segmentation methods. Full details of signal processing methods used.
(DOCX)

S1 Fig Frequency response of acoustic sensor developed for the FMM. (TIF)

S2 Fig Example signal exclusion by detecting maternal movements. (TIF)

S1 Table Data record. Spreadsheet of patient data.
(XLSX)
