# Supplementary material for: Performance of a wearable acoustic system for fetal movement discrimination
Source: PLoS One. 2018 May 7;13(5):e0195728. doi: 10.1371/journal.pone.0195728 (PMC5937742; doi:10.1371/journal.pone.0195728)
Supplement: S1 File — Full details of signal processing methods used. (DOCX) [file pone.0195728.s001.docx]

**Supplementary Data: Sensor Performance and Signal Analysis**

The signal processing software, with the full anonymised dataset, is freely available at <https://github.com/yalexand/Imperial-FMMtools>.

**Sensor Frequency Response**

A frequency sweep of the acoustic sensor was performed to assess its ranges of operation. An experimental set up consisting of a sheet of polymer acrylic glass placed over the sensor and attached to vibration table was designed as described in (22). The sensor response to a frequency sweep input from the table was recorded. Figure 1D shows the sensor response versus the input frequency. The sensor has a valid range of 1 Hz-1 KHz, with power concentrated below 50 Hz, which is significantly lower than accelerometers or open microphones.

**Figure S1**: Frequency response of acoustic sensor developed for the FMM. A vibration table with a sheet was place over the sensor and a sweep of 1Hz-1Khz was performed

**Signal Segmentation Architecture**

In order to identify a Region of Interest (ROI) vector consisting of ‘candidate movements’ for each scans, the following steps were applied to each record:

1. Each channel was assumed to be a random signal $\tilde{s}$ sampled at a frequency *f*. For the *i*-th channel, the transformed signal ${\tilde{s^{*}}}_{i}$ suitable for S/N analysis was created from $\tilde{s}$ as:

$${\tilde{s^{*}}}_{i}=\left| \tilde{s}_{i}-{<\tilde{s}_{i}>}_{fw} \right|$$

, where the denotation <..>*_R_* stands for the local signal averaging over the discrete range *R*.

Thus, ${\tilde{s^{*}}}_{i}$ is the absolute value of $\tilde{s}_{i}$ shifted by its zero mean over a time window of *w*. A value of w= 5 seconds was selected.

A noise estimate *e* was created from the statistical sample $\tilde{e^{*}}$, which was derived from $\tilde{s^{*}}$ as set of values in ${\tilde{s^{*}}}_{i}$not exceeding the lower quantile *q* of ${\tilde{s^{*}}}_{i}$:, that we will denote as$LQ(\mathop{s_{i}^{*}}\limits^{\sim} )$

, where *q* is the lower quantile of the signal assumed to be dominated by noise. *e* was taken as the median value of $\tilde{e^{*}}$ with a value of q=0.1.

1. For each channel *i* in subject test section, the binary ROI segmentation (detection) map $r_{i}$ was created by thresholding the signal above the level $h=le$ depending on the noise estimate *e*:

$$if {\tilde{s^{*}}}_{i}\geq h, r_{i}= {\tilde{s^{*}}}_{i}, else r_{i}=0$$

Values of l=150 and l=200 were used for acoustic and IMU channels respectively.

1. A temporo-spatial exclusion mask was applied to the acoustic sensor channel to eliminate signal artefacts when the probe was moved from the ROI. The mask was implemented to ensure that the system eliminated the ROIs detected when the probe was moved, so that it would not be selected in any subsequent fetal movements.

Segmented ROIs were dilated by 1.2 s by applying a binary dilation operation. That allowed joining together segmented signals, and provide enough signal length for ROI frequency analysis.

1. At the next stage, the strong signals (ROIs) segmented in IMU channels, detecting maternal motion, were checked for temporal overlap with any of the acoustic sensor channels’ ROIs segmented in the same session. If such overlap happened, any acoustic ROIs occurring during this overlap were discarded due to detection of maternal activity. Figure S2 shows an example set of acoustic signals where ROIs that appeared to be fetal movements were rejected by the system due to temporal overlap with maternal movement as detected by the accelerometer.

**Figure S2**: **Example signal exclusion by detecting maternal movements.**

Note the highlighted signals at 78 and 107 sec. These signals were excluded as being a result of maternal movement based on the output of the accelerometers. This fusion technique holds promise for future systems with multiple modes of sensing to reject the many potential physiological activities that may create false positives

1. At the next stage, the acoustic sensor ROIs were joined logically with an OR function to create a Single binary signal incorporating ROIs detected in all channels listening the events:

$$\hat{r}=\sum_{i=2}^{n} \tilde{r}_{i-1}\neg\tilde{r}_{i}$$

, where $\hat{r}$ is the final vector containing all ROI information. $\hat{r}$ was used for feature extraction such that it would not contain multiple signatures corresponding to the same fetal motion event

1. Finally, to reduce interference accumulated during the signal processing procedures, a minimal intensity criterion was applied to all channels using the absolute units of the signals’ amplitudes (0.05V for IMU and 0.004V for acoustic sensors). De-trended signals falling below these limits were disregarded. The remaining acoustic channel ROIs were deemed ‘candidate movements’.
