# Supplementary figures and images for: Performance of a wearable acoustic system for fetal movement discrimination
Source: PLoS One. 2018 May 7;13(5):e0195728. doi: 10.1371/journal.pone.0195728 (PMC5937742; doi:10.1371/journal.pone.0195728)

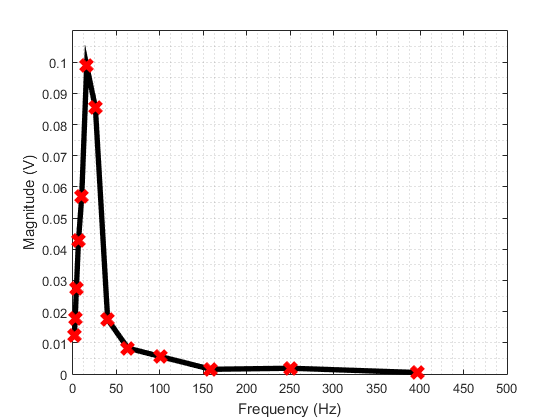

Supplement: S1 Fig — (TIF) [file pone.0195728.s002.tif]

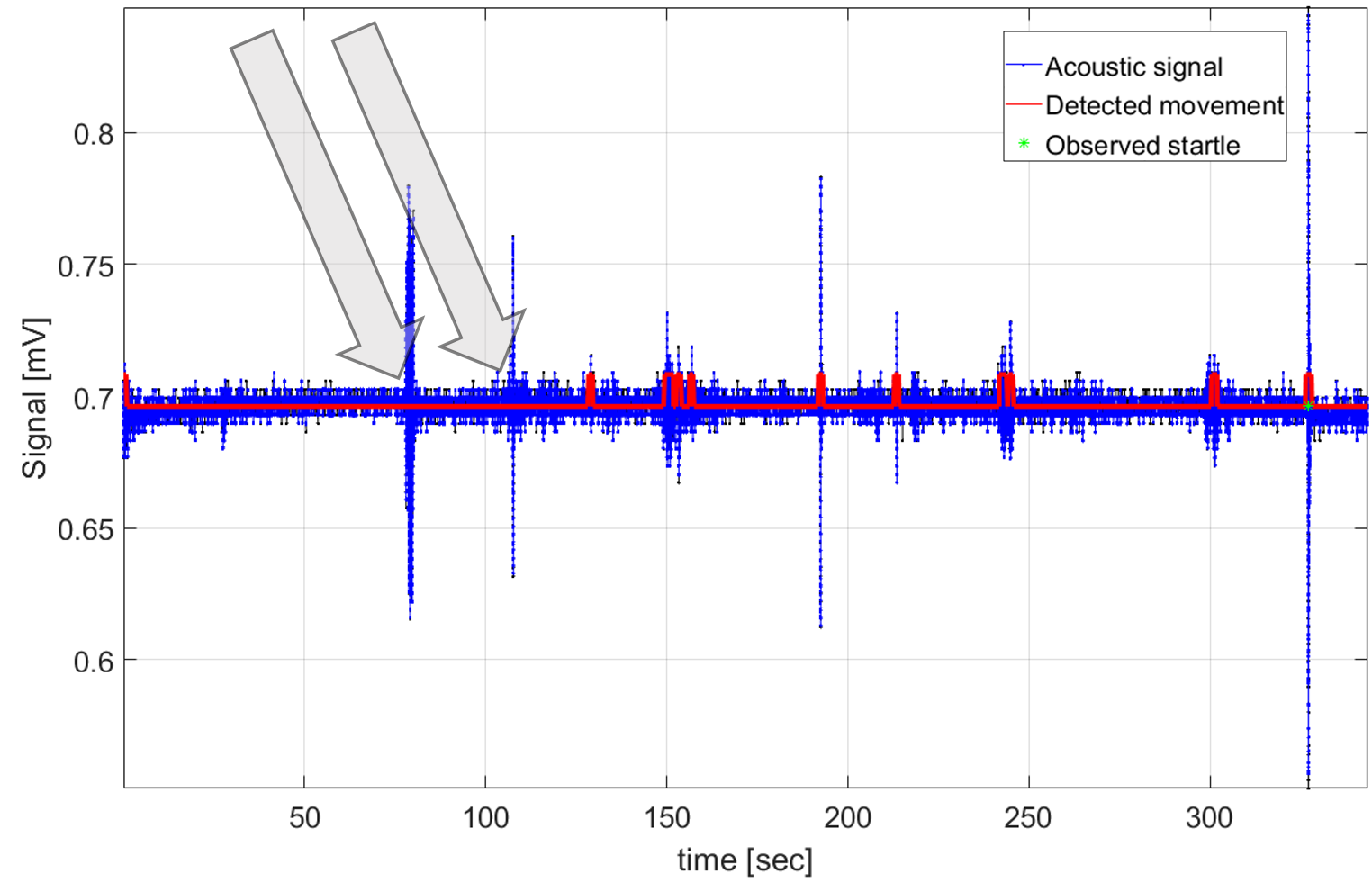

Supplement: S2 Fig — (TIF) [file pone.0195728.s003.tif]
